# Emerging Immunotherapies against Novel Molecular Targets in Breast Cancer

**DOI:** 10.3390/ijms22052433

**Published:** 2021-02-28

**Authors:** Vignesh Sivaganesh, Nazifa Promi, Salma Maher, Bela Peethambaran

**Affiliations:** 1 Department of Biological Sciences, University of the Sciences, 600 S 43rd St, Philadelphia, PA 19104, USA; vsivaganesh@mail.usciences.edu (V.S.); npromi@mail.usciences.edu (N.P.); smaher@mail.usciences.edu (S.M.); 2 Department of Biomedical Sciences, Philadelphia College of Osteopathic Medicine, 4170 City Ave, Philadelphia, PA 19131, USA

**Keywords:** immunotherapy, breast cancer, CAR-T, bispecific antibodies, mRNA vaccine, TNBC, triple-negative breast cancer, tumor specific/associated antigens, precision therapy, immune checkpoints

## Abstract

Immunotherapy is a highly emerging form of breast cancer therapy that enables clinicians to target cancers with specific receptor expression profiles. Two popular immunotherapeutic approaches involve chimeric antigen receptor-T cells (CAR-T) and bispecific antibodies (BsAb). Briefly mentioned in this review as well is the mRNA vaccine technology recently popularized by the COVID-19 vaccine. These forms of immunotherapy can highly select for the tumor target of interest to generate specific tumor lysis. Along with improvements in CAR-T, bispecific antibody engineering, and therapeutic administration, much research has been done on novel molecular targets that can especially be useful for triple-negative breast cancer (TNBC) immunotherapy. Combining emerging immunotherapeutics with tumor marker discovery sets the stage for highly targeted immunotherapy to be the future of cancer treatments. This review highlights the principles of CAR-T and BsAb therapy, improvements in CAR and BsAb engineering, and recently identified human breast cancer markers in the context of in vitro or in vivo CAR-T or BsAb treatment.

## 1. Introduction

Breast cancer is the second leading cause of cancer in women behind skin cancer [1]. Despite trailing behind skin cancer, the lifetime risk of developing breast cancer in females is 1 in 3, which currently leads all cancers [2]. In 2020, breast cancer will lead in new cancer diagnoses among women with an estimated 276,480 women and 2620 men diagnosed with invasive breast cancer [1]. Furthermore, 42,170 women are estimated to die from breast cancer, which is the second highest death total behind lung and bronchus cancer [1,2]. Though 5-year survival rates for stage 1 breast cancer are 85% or above, targeted therapy is needed for aggressive and highly metastatic triple-negative breast cancers that have a median survival of 10 to 13 months [2,3].

Current treatments for breast cancer vary depending on the expression of specific receptors on the surface of the cell. Breast cancer is classified based on expression of receptors which include estrogen receptor (ER), human epidermal growth factor (ERBB2) and progesterone receptor (PR). In general, breast cancer can be broken down into three major categories based on the combination of receptor expression: hormone receptor (ER or PR) positive/ERBB2 negative (HR+/ERBB2–), ERBB2 positive/hormone receptor positive or negative (ERBB2+/HR– or HR+), and triple-negative (HR–/ERBB2–) [3]. The hormone receptors (HR), after binding estrogen or progesterone, function as transcription factors to hundreds of genes implicated in cell division and growth [4]. To classify breast cancer as HR+, greater than 1% of the tumor must stain for either ER or PR [5]. To classify the tumor as ERBB2 positive, ERBB2, also known as HER2, must be overexpressed in the tumor cells [6]. ERBB2 is a 185 kDa transmembrane glycoprotein receptor in the epidermal growth factor (EGFR) family [7].

Activation of ERBB2 receptor triggers intracellular tyrosine kinase activity and a signaling cascade leading to cell survival and metastasis of cancer [8]. In addition to classification by receptor expression, breast cancer can also be classified into ductal or lobular carcinomas [3]. This histological classification depends on whether the cancer’s origin is within the ducts or lobules of the breast. However, classifying breast cancer as HR+/ERRB2–, ERBB2+/HR– or HR+, and triple-negative is vital for targeted therapeutics. 

Cancers that are HR+ in premenopausal women are treated with tamoxifen, while HR+ cancers in post-menopausal women are treated with aromatase inhibitors such as anastrozole, exemestane, and letrozole [3]. Tamoxifen is a selective estrogen receptor modulator (SERM) that acts as an estrogen receptor antagonist at the breast, preventing estrogen from binding and performing its normal function [3,9]. Aromatase inhibitors prevent conversion of androgen to estrogen and lower circulating estrogen levels [3,9]. In contrast to HR+ cancers, HR– cancers (ER and PR-) most likely promote cancerous growth and proliferation through alternate pathways. Inhibiting estrogen binding or lowering estrogen production will not be an effective form of treatment in HR– breast cancer. Therefore, treatment may have to target overexpressed ERBB2. Patients with ERBB2+/HR+ cancers are treated with a combination of anti-HR and anti-ERBB2 treatment [3]. Specifically, ERBB2 overexpressing cancers are treated with monoclonal antibodies such as trastuzumab and pertuzumab, which block endogenous ligand binding to ERBB2 receptors [3]. Alternatively, small molecule tyrosine kinase inhibitors such as lapatinib and neratinib can target the intracellular tyrosine kinase domain and inhibit the ERBB2 signaling cascade [3]. Unlike ERBB2+ cancers, those that do not overexpress ERBB2 promote anti-apoptosis and metastasis through other mechanisms and will not respond to ERBB2 inhibitor treatment. ERBB2–/HR– breast cancers are extremely challenging to treat since there are no well-known receptor targets that will slow down the proliferative and metastatic phenotypes of this cancer.

Cancers that are HR–/ERBB2– are referred to as triple-negative breast cancer (TNBC). Since ER, PR, and ERBB2 cannot be targeted for treatment, clinicians will use nonspecific chemotherapeutic agents for treatment [3]. HR+ and ERBB2+ stage 1 breast cancer have a 99% and 95% 5-year survival, respectively, while stage 1 TNBC has an 85% 5-year survival [10]. Metastatic HR+ and ERBB2+ breast cancer has a median overall survival of 4 to 5 years and 5 years, respectively, while metastatic TNBC has a median overall survival of 10 to 13 months [3]. It is evident that TNBC, especially metastatic TNBC, remains highly lethal. Therefore, it is imperative to find new therapies to treat TNBC. Recent studies have found new molecular targets in breast cancer that exhibit selectively lethality to TNBC in vitro and in vivo. This selective lethality arises because the tumor-associated antigen is either lowly expressed or not expressed in normal tissue. Moreover, scientists can harness the power of the immune system via chimeric antigen receptor-T cell therapy (CAR-T therapy) and bispecific antibody therapy (BsAb) to promote immune cell mediated death of cancer cells that highly express the target of interest. In this review, we will highlight emerging in vitro and in vivo CAR-T and BsAb therapy against novel tumor-associated or tumor-specific antigens.

## 2. Principles of Chimeric Antigen Receptor-T Cell (CAR-T) Therapy

**Figure 1:**
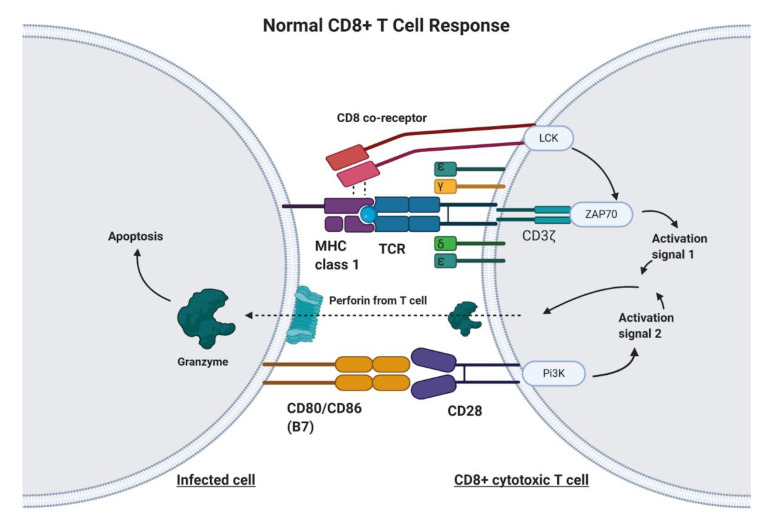
The Normal CD8+ T Cell Response. Infected cells will process and present a portion of the viral or bacterial protein on major histocompatibility complex (MHC) Class 1 molecules. The T cell receptor (TCR) on CD8+ T cells will recognize this antigen bound to MHC. The CD8 co-receptor helps with recognition of MHC Class I. Upon TCR/CD8 co-receptor binding, the intracellular CD8 and CD3ζ domain work together to produce activation signal 1. Additionally, a costimulatory signal involving B7 and CD28 is required in order to promote T cell activation, cytokine production, and prevention of anergy and apoptosis. Upon cytotoxic T cell activation, the T cell releases perforin and granzyme, which act upon the infected cell to initiate the extrinsic apoptosis pathway.

CAR-T cell therapy is designed to target any non-MHC (major histocompatibility complex protein), tumor-bound receptor and is based on the fundamental mechanisms of endogenous T cells. T cells are responsible for recognizing antigens from foreign material or harmful cellular activity, such as pathogens, environmental factors, and tumors (Figure 1) [11]. Antigen-presenting cells (APC), such as macrophages, dendritic cells, and B cells, process foreign invaders and present a piece of their proteins, known as antigen, to inactivated helper T cells via MHC class II [12]. The helper T cell will recognize this antigen with the help of its CD4 co-receptor, which assists in T cell receptor (TCR) binding to the antigen–MHC complex [12]. In addition to T cell receptor binding, the APC displays co-stimulatory proteins that bind to complementary receptors on the T cell to further enable its activation [12]. Upon activation, helper T cells facilitate in the activation of cytotoxic T cells, which are responsible for killing infected cells by binding to antigens presented on MHC Class I protein and inducing apoptosis (Figure 1) [12]. Unlike class II MHC proteins, which are restricted to dendritic cells, macrophages, and B lymphocytes, class I MHC proteins are found on most nucleated cells of the body [12]. T cell activation signaling through the TCR/CD3ζ pathway and through costimulatory receptors, along with much lower affinity for self-peptide-MHC (pMHC), allows for specific CD8+ T cell mediated cytotoxicity (Figure 1) [13].

**Figure 2:**
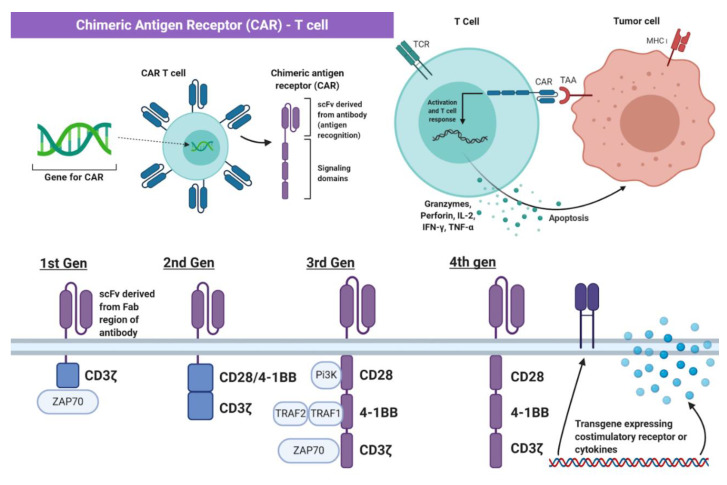
The Chimeric Antigen Receptor T cell (CAR-T Cell). The chimeric antigen receptor (CAR) construct is inserted into T cells and enables initiation of apoptosis via binding to tumor-specific or tumor-associated antigens independent of MHC interaction. Part of the figure adapted from “Chimeric Antigen Receptor (CAR)” and “CAR-Engrafted T cell and Tumor Cell”, by BioRender.com (2021). Retrieved from https://app.biorender.com/biorender-templates (accessed on 28 February 2021).

Genetic engineering has utilized the understanding of T cell recognition to create chimeric antigen receptors (CARs) that can be inserted into T cells for specific targeting and lysis [13]. The key component of the CAR is the extracellular single-chain variable fragment (scFv) derived from the fragment antigen binding (Fab) region of immunoglobulin, or another extracellular ligand recognition domain, which defines the T cell’s ability to bind and attack a specific molecular target independent of MHC protein [13]. In addition to the surface domain, CARs are composed of transmembrane and intracellular signaling domains [14]. Contained within these intracellular domains are CD3ζ, CD28, and 4-1BB [15,16]. Normally, T cell receptor heterodimers and CD3ζ join with CD3-gamma, CD3-delta, and CD3-epsilon to form the T cell receptor–CD3 complex [17]. However, the zeta chain specifically plays a role in coupling antigen recognition with several signal transduction pathways, which is the reason for its inclusion in CARs (Figure 1 and Figure 2). Another costimulatory receptor included in the CAR construct, CD28, is critical for T cell activation and operates through CD28 on the T cell interacting with CD80 or CD86 on APCs (Figure 1) [18,19]. CD28 engages the PI3K–AKT pathway to enhance T cell survival, growth, and clonal expansion (Figure 2) [19,20]. The final intracellular domain included in CARs is the 4-1BB costimulatory receptor, which amplifies and diversifies the T cell response after initial activation (Figure 2) [21]. Throughout time, more research and understanding of T cell and costimulatory receptors led to improvements in the CAR construct [22]. In first generation CARs, the scFv (single-chain variable fragment) is joined only by the intracellular CD3ζ domain and proved to be insufficient due to lack of lasting T cell response or sustained cytokine release (Figure 2) [23]. Second generation CAR constructs combined CD3ζ with another costimulatory domain (either CD28 or 4-1BB) to improve activation and survival of the CAR-T cells (Figure 2) [24]. The third generation CAR goes a step further to combine CD3ζ, CD28, and 4-1BB stimulatory domains (Figure 2) [25]. Finally, fourth generation CAR-T cells utilize the third generation CAR construct in addition to transgene expression of costimulatory receptors (CD28, 4-1BB, etc.) or proinflammatory cytokines (Figure 2) [25]. Fourth generation CAR-T cells are currently being heavily investigated and have shown great success in preclinical models [25]. Overall, the CAR is introduced into a patient’s own T cells to allow for maximal T cell activation and specific lysis of tumors via interaction with any tumor-associated or tumor-specific protein independent of MHC presentation [14].

Creating CAR-T cells from patients’ own leukocytes requires collection, separation, and genetic modification [14]. First, peripheral blood mononuclear cells are extracted from the patient and then the collected apheresis is washed and fractioned [26]. Different devices allow for removing red blood cell and platelet contamination, isolating lymphocytes through size-based fragmentation, and even further isolation of CD4+, CD8+, CD25+, or CD62L+ T cells using beads or chromatography techniques [26]. After the T cells are isolated, they are activated through multiple methods, such as APC or artificial APC based stimulation [26]. Additionally, products such as the Miltenyi MACS GMP TransAct CD3/28 beads, Miltenyi MACS GMP ExpAct Treg beads, Invitrogen CTS Dynabeads CD3/28, and the Juno Stage Expamer technology have been developed to activate and expand the T cells [26]. 

After collection and separation, the T cells are genetically modified to express the CAR construct through viral transfection, such as σ-retroviral vectors and lentiviral vectors [26]. Retroviral vector transfer utilizes the ability of the virus to convert a single-stranded RNA into a double-stranded DNA that then incorporates itself into the cell genome permanently through transduction [27]. One disadvantage of retroviral vectors is their inability to incorporate into nonreplicating cells [28]. This problem is solved by lentiviral vectors, which are capable of infecting dividing and nondividing cells [29]. Another stable vector comprises the sleeping beauty transposon packaged into a lipid-based vessel [26]. In this system, a transposase enzyme, generated by intracellular machinery transcribing the expression vector then translating to the enzyme, recognizes inverted tandem repeat sequences flanking the transgene on the other vector and then cuts and pastes the gene into TA dinucleotide base pair sites of the T cell genome [30]. 

Additionally, other methods of generating CAR-T cells have also been developed. Researchers have worked on a protocol involving mRNA electroporation to generate CAR-T cells, which can prevent uncontrolled reactivity caused by viral transductions [31]. Scientists have also used CRISPR to successfully integrate a CD19-specific CAR into the T cell receptor α constant (TRAC) locus, which allowed for robust expression of the CAR and outperformed the function of CARs generated by other methods [32].

In comparison to conventional T cell activity, challenges with safety and specificity arise in CAR-T cells [13]. Unlike conventional T cells, which have a high specificity for their targets, CARs often target both the cells of interest and healthy cells due to the unrestricted nature of certain targets [13]. Researchers have aimed to address this by engineering CAR-T cells that can be eliminated either through conditional suicide gene insertions or through transduced caspase-9, which induces apoptosis upon AP1903 drug administration [13]. It is also possible to target molecules expressed on tumor cells but absent or lowly expressed on healthy cells, such as ROR1 or GD2, or neoantigens present due to oncogenic mutation [13]. An alternative solution could be to transduce T cells with both a CAR and a chimeric costimulatory receptor (CCR) that recognize separate antigens, therefore requiring recognition of two tumor-associated proteins before CAR-T cell activation [33]. Through advanced development of different generations of CAR-T constructs as well as improvements in specificity and activation, CAR-T therapy will undoubtedly be a key component of future targeted immunotherapeutics.

## 3. Principles of Bispecific Antibody Therapy

**Figure 3:**
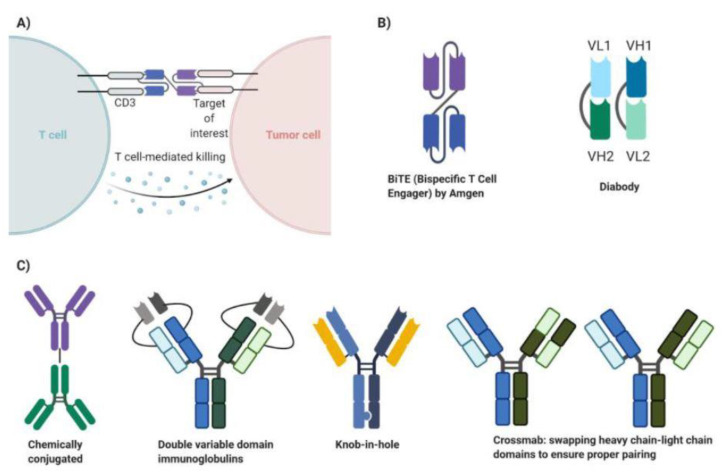
Bispecific antibodies. Bispecific antibodies (BsAbs) bind to the tumor target of interest and induce T cell activation, localization to tumor, and polyclonal proliferation by simultaneous interaction with the T cell’s CD3 domain (A). The bispecific antibody’s function arises from its ability to bind two separate antigens (**A**). Various bispecific antibody structures exist as illustrated (**B**,**C**). Part A of the figure adapted from “Bispecific Antibody Design”, by BioRender.com (2021). Retrieved from https://app.biorender.com/biorender-templates (accessed on 28 February 2021).

Bispecific antibodies (BsAbs), though there are many variants, are generally constructed with two different fragment antigen-binding (Fab) regions. One Fab region binds to one tumor receptor, while the other region binds to another tumor receptor, enabling the BsAb to inhibit the function of two tumor receptors that could be important in growth, proliferation, angiogenesis, or metastasis [34]. Alternatively, one Fab region of the BsAb will bind to a tumor receptor, while the other region will bind to the CD3 domain of T cells, resulting in localization, polyclonal proliferation, and specific lysis of the tumor via CD8+ T cells (Figure 3A) [35]. Initially, bispecific antibodies were either generated by chemical conjugation of two separate IgG monoclonal antibodies or by fusing hybridomas (immune cells fused with myeloma cells) to generate a two heavy chain, two light chain containing quadroma (Figure 3C) [34]. The generation of quadromas would mean that 10 different IgG molecules would be produced, with only one of them having the correct pairing to bind to the two separate antigens of interest [36].

To overcome inefficient heavy chain–light chain pairing, scientists have engineered different ways to promote correct pairing. Double variable domain immunoglobulins have the two variable light chains linked together within the light chain construct and two variable heavy chains linked together within the heavy chain construct (Figure 3C) [34]. This means that only one heavy chain and one light chain gene exist, generating one tetrameric immunoglobulin capable of binding two different antigens. Alternatively, the knob-in-hole technique was created to ensure proper heavy chain pairing. A mutation introduced on the constant domain of one heavy chain would produce a larger side chain (the knob), while a mutation on the constant domain of the other heavy chain would produce a smaller side chain known as the hole (Figure 3C) [37]. This would ensure that the two distinct heavy chains would properly pair, but the two distinct light chains could still mispair with the heavy chains. Therefore, certain heavy chain domains would be swapped with the corresponding light chain domains to ensure correct pairing (Figure 3C) [34]. This technique, called crossmab, allows for the correct heavy chain–light chain pairing to occur. Bispecific antibodies generated by the aforementioned techniques have the fragment crystallizable region (Fc), which promotes antibody-dependent cellular cytotoxicity, longer half-life, and complement fixation in addition to specific targeting of the molecules of interest [34].

Recent advancements in genetic recombination techniques have enabled scientists to generate bispecific antibodies that contain only the Fab regions (Figure 3B). These bispecific antibodies can be generated from a single genetic sequence, bypassing any concerns about proper heavy chain–light chain pairing. However, these constructs lack the Fc domains, meaning their mechanism of action only arises from dual-receptor inhibition or recruitment of T cells to the specific tumor target [34]. One popular bispecific antibody construct lacking the Fc portion is the bispecific T cell engager (BiTE), developed by Amgen (Figure 3B). The BiTE is composed of two single-chain variable fragments (scFv) [38]. The scFv consists of a heavy chain and light chain fused by a linker sequence [38]. Two scFvs, each with the ability to bind to a unique antigen, are then fused by a linker sequence to generate the BiTE (Figure 3B) [38]. An alternative to BiTE is the diabody bispecific antibody (Figure 3B). This construct is made from two polypeptide chains, each containing the variable heavy chain unique to one antigen and the variable light chain unique to the other antigen [34]. This format forces the correct heavy chain–light chain pairing to generate a diabody that can bind two unique antigens (Figure 3B) [34]. Furthermore, bispecific antibodies lacking the Fc portion can be conjugated to proteins that can provide an increased half-life or extra functionality [34]. Overall, bispecific antibodies can generate specific antitumor responses via activation of CD8+ T cells and, unlike CAR-T therapy, do not require the extraction, genetic modification, and reinsertion of patient T cells. There are many more bispecific antibody constructs beyond the previously described Fc-containing immunoglobulin bispecifics and Fab-only bispecific antibodies. We have highlighted the most important advancements in bispecific antibody development and some popular bispecific antibody formats in this review.

## 4. Development of CAR-T Cell Therapy in Breast Cancer

The advent of chimeric antigen receptors has made it possible to explore alternative targets for cancer therapy, especially in TNBC that lacks the common therapeutic targets ERBB2 and HR. One highly selective target in TNBC, MUC1, arises as a result of overexpression and post-translational modifications [39]. MUC1 is a glycosylated transmembrane protein normally expressed on luminal and glandular epithelium, including the breast [39]. Normally, MUC1 produces mucin on the apical surfaces of epithelium, which function as a protective barrier against pathogens [40,41,42]. As malignant transformation occurs, MUC1 becomes highly overexpressed and hypoglycosylated, leading to the activation of intracellular signaling pathways that promote cancerous phenotypes [39,42]. The hypoglycosylation of MUC1 also leads to the exposure of protein epitopes that are absent in normal, heavily glycosylated MUC1 [43]. Previous studies have shown that an antibody known as TAB004 specifically binds to tumor specific MUC1 (tMUC1) [44]. This led to the construction of CAR-T cells that contain the extracellular scFV derived from TAB004 [39]. CAR-T cells against tMUC1, known as MUC28ζ CAR T cells, effectively recruited peripheral blood lymphocytes (PBLs), increased expression of leukocyte activation markers, and promoted cytokine production in vitro [39]. Furthermore, MUC28ζ CAR T cells promoted extensive cancer cell lysis in a wide range of TNBC cell lines in vitro [39]. A single injection of MUC28ζ CAR T cells showed long-term effects in reducing tumor growth in vivo [39]. A recent analysis of 5861 breast cancer patients showed that MUC1 overexpression has predictive value and is associated with poor clinical outcomes [45]. Given this information, targeting the emerging tumor-specific antigen tMUC with immunotherapy shows great promise in breast cancer.

CAR-T cell therapy against integrins has also been explored. Integrins are cell surface receptors that promote migration, invasion, survival, and proliferation and are known to be highly expressed in breast cancer [46]. The known functions and increased expression of integrin led to studies targeting tumor-associated integrins. CAR-T cell treatment against αvβ3 integrin showed cytolytic effects against a range of in vitro cancers, including MDA-MB-231 TNBC cell lines [47]. Additionally, treatment with CAR-T cells proved to almost completely eradicate metastatic melanoma in mice treated with high affinity and low affinity CAR constructs against αvβ3 [47]. Previous αvβ3 CAR-T cell in vivo therapy reported selective cytotoxicity against αvβ3 expressing HUVEC cell lines while reporting no damage to normal tissue [48]. Clinical trials utilizing monoclonal antibody (mAb) against αvβ3 expressing angiogenic blood vessels or cancers did not report significant adverse effects [49,50]. While increased αvβ3 expression and in vitro cytolytic activity was reported in MDA-MB-231 breast cancer cells upon CAR-T therapy, more studies must be done to confirm that αvβ3 is a promising tumor-associated target for breast cancer immunotherapy.

Another target known as TEM8 (tumor endothelial marker), or ANTXR1, is an integrin-like protein known to be highly expressed in endothelium during endothelial cell development in mice but is minimally detectable in adult mice [51,52]. Furthermore, TEM8 is upregulated within the endothelium of various tumors in both humans and mice [53,54]. TEM8 expression is increased in invasive breast cancer and is associated with breast cancer relapse [55,56]. Specifically, TEM8 expression was identified in six TNBC cell lines and was especially elevated in Hs578T and MDA-MB-231 cell lines [57]. L2 CAR-T cells, derived from the L2 mAb that targets TEM8, proved to eradicate TNBC in vitro and caused significant tumor regression in vivo and in mice containing TNBC patient-derived xenografts [57]. A single dose of L2 CAR-T cell therapy also improved overall survival outcomes in TNBC patient-derived xenograft mice in comparison to control treatments [57]. TEM8 has great potential to be a possible tumor-specific target for CAR-T therapy against TNBC. 

ROR1 is another emerging target in breast cancer and other cancer types. ROR1, known as receptor tyrosine kinase-like orphan receptor 1, is a receptor highly expressed during embryogenesis and lowly expressed in adult tissue [58]. However, ROR1 expression is significantly increased in aggressive breast cancer and is associated with poor clinical outcomes [58]. In vitro, ROR1 expression is associated with TNBC cell lines MDA-MB-231 and MDA-MB-468 but is lowly expressed in MCF7 ER+ breast cancer cell lines [58]. Studies showed that ROR1 CAR-T therapy effectively killed MDA-MB-231 in microphysiologic 3D tumor models and generated a 20- to 30-fold increase in cancer cell apoptosis [59]. Furthermore, the ROR1 CAR-T cells were able to promote significant IL-2 and IFN-γ production and successfully penetrated and proliferated within the 3D tumor model [59]. Overall, tMUC, αvβ3, TEM8, and ROR1 have been successfully targeted by CAR T cells, which will hopefully stimulate intense research into other tumor-specific or tumor-associated antigens that can be targeted by immunotherapy (Table 1).

## 5. Development of Bispecific Antibody Therapy in Breast Cancer

Bispecific antibodies and CAR-T cells have been developed for ERBB2 (also known as HER) overexpressing breast tumors and have been tested in vitro, in vivo, and in clinical trials [60,61]. Though many clinical trials have been successful with no adverse reactions reported, one patient developed cardiopulmonary failure from excessive T cell activation, which most likely targeted both cancerous and noncancerous ERBB2+ expressing cells [62]. Recall that the definition of ERBB2+ breast cancer is an overexpression of ERBB2 receptor. Specifically, ERBB2+ breast cancer can have 25 to 50 copies of the ERBB2 gene, resulting in a 40- to-100-fold increase in ERBB2 receptor expression in comparison to normal cells, hence why ERBB2 is targeted by anti-ERBB2 antibodies and, more recently, immunotherapy [63,64]. However, it is possible that better targets may exist since ERBB2 is still expressed in normal tissue and can lead to adverse effects as described in the patient with cardiopulmonary arrest. One potential tumor-specific antigen, p95HER2, is a truncated version of HER2 expressed in 40% of HER2+ breast cancers and not expressed in normal adult tissue [65]. One study found that selectively targeting p95HER2 positive cells with CD3-p95HER2 BsAb (CD3-p95HER2 describes the two targeted antigens in this BsAb) was cytotoxic to cancer cells expressing p95HER2 and was nonlethal to HER2+/p95HER2- tissue in vitro and in vivo [65]. Additionally, CD3-p95HER2 BsAb did not lead to the activation of the T cell response against non-transfected MCF10A breast epithelial cells, which contrasts with the significantly elevated recruitment and activation of T cells with HER2 BsAb treatment [65]. The CD3-p95HER2 BsAb was almost completely nonlethal to normal tissue and has potential to cause highly specific toxicity of p95HER2+/HER2+ breast cancer in humans [65]. These results are encouraging and may be one possible tumor-specific target in HER2+ breast cancer. Since p95HER2 is a truncated HER2 receptor generated by alternative translation initiation of the normal HER2 mRNA transcript, other targets must be found in TNBC that lacks HER2, estrogen, and progesterone receptor expression.

One possible molecular target is the vascular endothelial growth factor receptor 1 (VEGFR1), a receptor tyrosine kinase that binds various VEGF and placental growth factors to promote migration and survival of hematopoietic stem cells and leukemia cells [66]. Though VEGFR1 is expressed in endothelial cells, most endothelial cell functions are mediated by VEGFR2 [66]. VEGFR1 overexpression has been strongly linked to increased proliferation, invasion, and migration in breast cancer [67,68]. Therefore, VEGFR1 may be a useful tumor-associated antigen candidate for BsAb therapy. One study discovered that utilization of VEGFR1-CD3 BsAb induced proliferation of in vitro peripheral blood lymphocytes (PBL) and increased PBL CD25 expression similarly to OKT3 (functional anti-CD3 antibody) activated PBLs [68]. Furthermore, VEGFR1-CD3 BsAb promoted selective cytotoxicity of MDA-MB-231 and MDA-MB-435 TNBC cell lines when co-cultured with PBLs, while being almost perfectly nonlethal to HEK-293 cells even at high effector-to-target ratios [68]. VEGFR1-CD3 BsAb shows promising results against VEGFR1 expressing breast cancer cell lines. Most endothelial cell functions are mediated by VEGFR2, which may possibly indicate that VEGFR1 inhibition is nonlethal to normal endothelial cells. Still, further investigation is required to ensure high selectivity towards VEGFR1 expressing cancers and retention of normal endothelial cell phenotype and function. VEGFR1 may be a strong molecular target for TNBC immunotherapy due to its heightened activity in cancer and minimal activity in endothelial cells.

Another possible tumor-associated antigen for TNBC therapy is prolactin receptor (PRLR). Prolactin receptor’s ligand, prolactin, is an important hormone generated by the pituitary gland and functions to produce milk and further develop the mammary glands by binding to PRLR within breast tissue [69]. Prolactin receptor is highly expressed in certain breast cancer subtypes [70]. In comparison to normal breast epithelium, PRLR is expressed slightly more in the MDA-MB-231 TNBC cell line and is significantly more expressed in SKBR-3 and T47D HER2+ cell lines [70]. Previous reports utilized a bispecific antibody against HER2 and PRLR to enhance cytotoxicity when combined with HER2 antibody drug conjugate and when the bispecific antibody itself was conjugated to drug [71]. In this case, the mechanism of BsAb-induced cytotoxicity arose from inhibition of HER2 and PRLR simultaneously, rather than recruitment of cytotoxic T cells via a CD3 binding domain [71]. Furthermore, cytotoxicity occurred from the delivery of the drug to its intended target [71]. While simultaneous PRLR and HER2 inhibition has been investigated, further studies have been done to assess PRLR alone as a viable target for TNBC. One study showed that a CD3-PRLR BsAb enhanced cytotoxicity against MDA-MB-231 in comparison to PRLR mAb treatment [70]. Furthermore, CD3-PRLR BsAb recruited CD4+ and CD8+ T cells similarly to CD3 mAb, but markedly increased cytokine production in comparison to CD3 mAb [70]. Though these initial results are promising, future studies must be done to show that CD3-PRLR BsAb does not cause unintended cytotoxicity to normal prostate, ovary, adipocyte, and liver tissue, all which have significant PRLR expression [72]. A study involving 73 patients, 53 of which were treated at the highest dose of PRLR mAb, concluded that there were no dose-limiting toxicities [73]. However, BsAb treatment is known to be more lethal, thus requiring more investigation into the toxicities of BsAb treatment against a variety of normal tissue. The CD3-PRLR BsAb may be an effective alternative to VEGFR1-CD3 BsAb and could possibly be combined with VEGFR1-CD3 BsAb therapy in future in vitro and in vivo studies.

In addition to BsAb-mediated therapy alone, a cocktail of BsAbs can be utilized to promote CAR-T cell-induced cytotoxicity of cancer cells. This form of therapy allows different BsAbs to recruit a single population of CAR-T cells, inducing cytotoxicity of metastatic tumors located throughout the body that may express different antigens due to mutations. For example, a group of researchers constructed CAR-T cells with an external scFv capable of binding to fluorescein (FITC) [74]. Then, they created three different BsAbs: FITC-folate (FRα ligand), FITC-DUPA (prostate specific membrane antigen ligand), and FITC-AZA (acetazolamide inhibits carbonic anhydrase) [74]. Folate receptor overexpression is associated with breast cancer, while prostate specific membrane antigen (PSMA) and carbonic anhydrase (CA) IX overexpression is most associated with prostate and renal cell cancer, respectively [75,76,77]. Yet, researchers wanted to identify whether a cocktail of BsAbs could recruit a single population of T cells to simultaneously target multiple tumor-associated antigens. In separate experiments, FITC-folate, FITC-DUPA, and FITC-AZA BsAb treatment with FITC CAR-T cells reduced the tumor volume of in vivo MDA-MB-231 cells overexpressing FRα, PSMA, and CA IX, respectively [74]. In another experiment, FITC-DUPA and FITC-AZA combination treatment significantly reduced tumor volume of two distinctly located subcutaneous MDA-MB-231 cell lines within the same mouse, one overexpressing CA IX and the other overexpressing PSMA [74]. Furthermore, the combination treatment of FITC-DUPA and FITC-AZA promoted cytotoxicity of a single in vivo MDA-MB-231 tumor overexpressing PSMA and CA IX in the presence of FITC CAR-T cells in comparison to FITC-DUPA and FITC-AZA treatment alone [74]. Overall, though this BsAb cocktail and CAR T-cell combination therapy shows remarkable promise as a future therapeutic for isolated tumors or multiple metastatic tumors with antigenic variability, further studies are required to ensure that this form of therapy works on tumors that naturally possess heterogeneity.

Folate receptor transports folate into cells to function in the DNA synthesis biochemical pathway [75]. One study identified significant enrichment of folate receptor mRNA and protein in TNBC cell lines compared to ER+ and HER2+ breast cancer cell lines [75]. The FITC-folate BsAb can potentially target cancer cells with moderate or increased expression of FRα as previously described. Prostate specific membrane antigen (PSMA) is a cell surface receptor lowly expressed in normal prostate and highly expressed in prostate cancer [76,78]. PSMA can convert Poly-γ-glutamate folate, released from dead and dying adjacent tumor cells, into folate, which can be taken up by the cell to promote DNA synthesis [78]. DUPA, a glutamate urea ligand, has high affinity for the PSMA receptor, thus can be included in a BsAb to target PSMA expressing cancers as previously described [76]. Carbonic anhydrase, specifically isoform CA IX, is a tumor-associated cell surface glycoprotein that is highly expressed in renal cell cancers [77]. Increased CA IX arises from hypoxic conditions induced by rapid tumor outgrowth of its blood supply [77,79]. CA IX catalyzes the formation of H+ in the extracellular space by converting carbon dioxide and water into hydrogen and bicarbonate [77,80]. Bicarbonate is transported into the cell while hydrogen promotes the reduction in pH extracellularly [79]. This reduction in pH promotes a migratory phenotype in cancers [77,81]. Acetazolamide (AZA) is a noncompetitive, reversible inhibitor of carbonic anhydrase that can be utilized on a BsAb as previously described [82]. Though only folate receptor is relevant to TNBC in the previously mentioned study, we included PSMA and CA-IX in order to provide insight into cocktail BsAb therapy capable of simultaneously targeting different receptors with unique functions. Cancers that exhibit molecular heterogeneity, through expression of multiple tumor-associated receptors, can be successfully targeted by combining a single population of CAR-T cells with a variety of BsAbs that target multiple cancer specific receptors. In particular, successful experimentation of BsAbs against p95HER2, VEGFR1, PRLR, and FRα warrants more robust research and possible BsAb cocktail therapy targeting varying combinations of these antigens (Table 2).

## 6. Improving Immunotherapy by Modulating Immune Checkpoint, Transforming Growth Factor (TGF), and IL-7R Signaling

While BsAb and CAR-T cell therapy promotes targeted therapy, the tumor microenvironment has intrinsic properties capable of suppressing the immune response. Normally, immune cells will upregulate expression of inhibitory receptors, also known as immune checkpoints, under chronic inflammatory conditions as a physiological mechanism to modulate the immune response and prevent autoimmune attacks [83]. Immune cells that have significant expression of these receptors are known as exhausted immune cells [83]. Regulatory T cells (Tregs), which constitutively express inhibitory receptors, can also play a role in immune suppression. Examples of immune checkpoints expressed on immune cells include programmed cell death protein 1 (PD-1), cytotoxic T-lymphocyte-associated protein 4 (CTLA-4), T cell immunoglobulin and mucin domain containing protein 3 (TIM-3), lymphocyte-activation protein 3 (LAG-3), and T cell immunoreceptor with immunoglobulin and ITIM domains protein (TIGIT). Tumors can upregulate expression of immune checkpoint ligands, which suppresses immune-based attack on the cancer [83]. In addition to inhibitory receptor expression increasing on immune cells as the immune response progresses, tumors can secrete a variety of chemotactic factors that will recruit CTLA-4 expressing Tregs [84]. CTLA-4 will bind to CD 80 or CD 86 receptor (also known as B7) on antigen presenting cells (APCs) with higher affinity than the CD28 receptor on CD4+ and CD8+ T cells, which will suppress costimulatory signals and inhibit activation of the helper and cytotoxic T cell response against the tumor [83,85]. Therefore, in order to improve the efficacy of immunotherapy, dual therapy with immune checkpoint blockers can promote a stronger antitumor response.

CAR-T cells, much like normal T cells, can express the PD-1 inhibitory checkpoint as the immune response progresses (Table 3). In addition to innate immune cells such as macrophages and dendritic cells, tumor cells can express high levels of the PD-1 ligand, PD-L1 [86]. Once tumors bind to PD-1 on CAR-T cells, the intracellular signaling pathway reduces pro-survival gene expression within the BCL family, cytokine production, and cell cycle progression [83,86,87,88,89]. Anti-PD-1 and anti-PD-L1 antibody treatments have been explored in clinical trials [90]. Particularly, anti-PD-1 antibodies can successfully block the PD-1–PD-L1 axis but do not have favorable clinical outcomes, possibly due to antibody capture by PD-1 tumor-associated macrophages [91,92]. This limitation can be overcome by implementing other strategies. For example, researchers used CRISPR-CAS9-mediated PD-1 knockout CAR-T cells against mesothelin, a glycosylphosphatidylinositol- anchored cell surface receptor that is overexpressed in TNBC and contributes to tumor invasion, metastasis, and drug resistance (Table 1; Table 3) [92]. PD-1 edited CAR-T cells against BT549 cells expressing mesothelin showed specific cytotoxicity and increased cytokine production in comparison to unedited mesothelin CAR-T cells and combined anti-PD-1 antibody treatment, both in vitro and in vivo [92].

**Figure 4:**
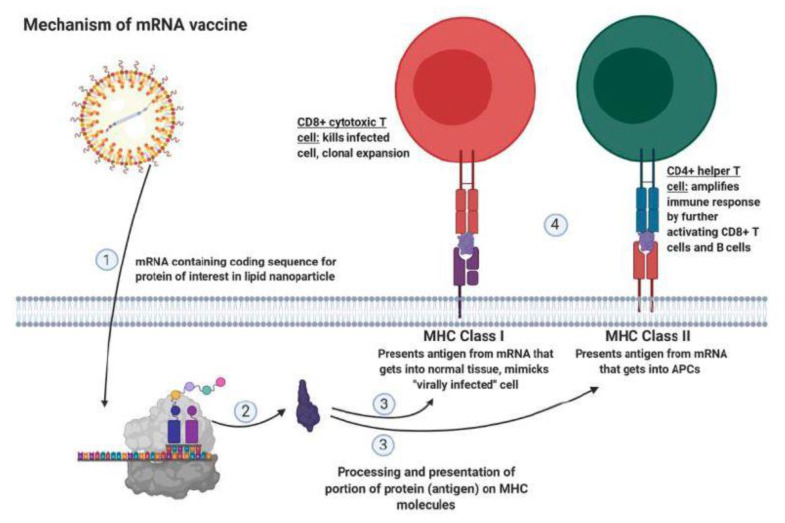
Mechanism of mRNA vaccine utilized in cancer therapeutics and COVID-19 prevention. The mRNA containing the coding sequence for the target protein is protected by lipid nanoparticles in order to ensure proper delivery to cells (1). Once the mRNA is inside cells, the intracellular machinery will translate the mRNA into the protein of interest (2). Then, this protein is processed as a foreign invader, which enables portions of the protein (antigens) to be presented on MHC class I by nucleated cells or class 2 by antigen presenting cells (3). MHC class I presentation generates killing of the cell as well as clonal expansion of the CD8+ T cell (4). MHC class II presentation induces the CD4+ helper T cell-dependent amplification of the immune response by further activating T cells and antibody secreting B cells (4). This mechanism, popularized by the recent invention of COVID-19 vaccines, has been previously utilized in cancer immunotherapy.

T cells are also susceptible to increases in CTLA-4 expression as the immune response progresses (Table 3) [93]. CTLA-4 has a higher affinity for B7 expressed on APCs [83]. Normally, B7 will bind to the CD28 costimulatory receptor on T cells and activate the T cell response in combination with T cell receptor binding to the correct antigen [93]. When CTLA-4 expression increases on T cells or Tregs, CTLA-4 will bind to B7 with higher affinity than CD28, inhibiting T cell activation [83]. Research has shown that targeting the previously described MUC1 receptor through mRNA vaccine in combination with CTLA-4 inhibition produced a robust antitumor response against TNBC [93]. Specifically, MUC1 mRNA vaccine delivery to dendritic cells within mice lymph nodes promoted cytotoxicity of MUC1+ TNBC in vivo [93]. The mRNA vaccine technology has recently been popularized due to its usage in the COVID-19 vaccine [94]. However, this technique has been utilized extensively prior to the advent of the COVID-19 vaccine for targeting tumor receptors in cancer therapy. The mRNA sequence is packaged into lipid nanoparticles, which allows for delivery of the mRNA sequence into cells without harming the construct (Figure 4) [93,95]. Once in the cell, the intracellular machinery will generate the protein of interest from the mRNA (Figure 4) [93,95]. In the context of cancer therapeutics, the generated protein will be taken up and processed by APCs or normal tissue, then presented on MHC class II or class I molecules to CD4+ and CD8+ T cells, respectively (Figure 4) [96]. The CD4+ T cells will recognize this protein as foreign and mount an immune attack by amplifying the CD8+ T cell and B cell response (Figure 4) [95,96]. This immune pathway normally occurs when the immune system recognizes a tumor receptor. However, delivering a mRNA vaccine greatly amplifies the immune response as shown by aforementioned study [93]. Furthermore, the addition of anti-CTLA-4 antibodies enhanced mRNA vaccine-induced cytotoxicity, CD8+ T cell tumor infiltration, and long-term reduction in tumor growth [93]. These results must be further explored with BsAb and CAR-T cell therapies (Table 3).

Another inhibitory receptor, LAG-3, binds to MHC class II molecules on APCs to elicit apoptosis and decreased proliferation of T cells [97,98]. Previous studies showed that anti-LAG-3 antibodies increased CD8+ T cell effector capabilities and decreased tumor growth in vivo (Table 3) [99]. In addition to LAG-3, TIM-3 is another immune checkpoint implicated in reducing the immune response in chronic inflammation and tumor infiltrating leukocytes [100]. TIM-3 binds galectin-9 released from myeloid-derived suppressor cells and leads to apoptosis of exhausted effector T cells [100,101]. Anti-TIM-3 antibody therapy proved to increase IFN-γ production and increased the population of tumor infiltrating CD8+ T cells in vivo (Table 3) [102]. Anti-LAG-3 and anti-TIM3 antibodies in combination with CAR-T or BsAb therapy have not been robustly researched in cancer immunotherapy and must be explored especially in TNBC.

TIGIT is a type 1 transmembrane receptor expressed on exhausted effector and memory T cells. TIGIT has an extracellular domain capable of binding to the poliovirus receptor (PVR) [103]. PVR, initially named due to its ability to mediate poliovirus entry into cells, has recently emerged as a ligand of TIGIT that reduces T cell proliferation and cytokine production upon binding [104]. One study utilized BsAb therapy against EGFR in combination with blocking the PVR–TIGIT axis with anti-TIGIT-antibody (Table 3) [103]. EGFR is a transmembrane glycoprotein overexpressed in cancer that functions in proliferation, modulation of adhesion, anti-apoptosis, invasion, and angiogenesis [103,105,106]. The study showed that this form of dual therapy increased production of CD8+ T cell granzyme and increased T cell induced cytotoxicity of TNBC cell lines [103]. While PD-1, CTLA-4, and TIGIT inhibition has been explored in the context of CAR-T, mRNA vaccine, and BsAb therapy, respectively, TIM-3 and LAG-3 require further investigation in immunotherapies that amplify the immune response. Furthermore, utilizing CRISPR technology to knock out immune inhibitory receptors, as performed in the PD-1 knockout mesothelin CAR-T cells, should be further explored to determine if knockout increases antitumor function in comparison to antibody-based inhibition of immune checkpoints. Combination therapy involving blocking one or more immune checkpoints along with BsAb or CAR-T therapy must be robustly investigated in vivo to assess efficacy under physiologic conditions.

Based on the data from current studies, it is evident that the future of immunotherapy may involve incorporating BsAb, CAR-T, or mRNA vaccine therapy in combination with immune checkpoint inhibition. However, there are other signaling pathways that can be manipulated to enhance the antitumor response of T cells. Immune stimulatory cytokines such as IL-7 are important in the recruitment, proliferation, and anti-apoptosis of T cells [107]. One study utilized CAR-T cells modified to express constitutively active IL-7 receptor and proved that these fourth-generation CAR-T cells against AXL, a receptor tyrosine kinase overexpressed in cancer and implicated in cell growth and survival, enhanced specific tumor lysis and cytokine production of IL-2, IL-4, IL-6, TNF-alpha, and IFN-γ in vivo (Figure 2; Table 1; Table 3) [108,109]. Another study focused on inhibiting the transforming growth factor beta (TGF-β) pathway in immune cells [110]. TGF-β binds to the TGF-β receptor, phosphorylating SMAD2 and SMAD3 and inducing the formation of the SMAD2-SMAD3-SMAD4 complex that enters the nucleus to bind to transcription factors [110]. These transcription factor complexes will regulate the production of many genes that generally inhibit the functions of the immune cell [110]. CAR-T therapy against ROR1 in the presence of TGF-β showed reduction in tumor lysis and cytokine production [110]. Inhibiting the TGF-β receptor with specific kinase inhibitor SD-208 enhanced tumor lysis and cytokine production in comparison to both TGF treated and untreated CAR-T conditions [110]. Both IL-7 and TGF-β modulation prove to significantly enhance CAR-T therapy and should be utilized in future immunotherapy studies (Table 3).

## 7. Concluding Remarks

Immunotherapy has come a long way throughout the years. CAR constructs have been continuously improved over time with the construction of three generations of CARs, with extensive research into the fourth generation CAR well underway (Figure 2). There are also many different versions of bispecific antibodies that all have slightly different pros and cons, but overall create a new wave of immunotherapy that does not require T cell extraction and reinsertion into a patient. However, the biggest improvement in immunotherapy has been the discovery of selective cancer receptors, which is especially important in TNBC that lacks the key proteins that are currently targeted in clinical therapy. A wide range of molecular targets have been investigated in the context of CAR-T and BsAb therapy. A few of the explored molecular markers are purely specific to breast cancer. However, tumor-associated antigens also show great promise since CAR-T and BsAb therapy can specifically target the tumor while having minimal cytotoxicity to normal tissue. Still, further research is needed for selective markers to ensure that these forms of immunotherapy have minimal toxicity to normal tissue, especially in the wake of inhibiting immune checkpoints or modulation of stimulatory and inhibitory signaling pathways to promote a robust T cell response. Perhaps the future of immunotherapy may involve a cocktail of bispecific antibodies for metastatic tumors that may vary in antigen expression due to mutations. Alternatively, for tumor-associated antigens that may cause significant cytotoxic effects in normal tissue minimally expressing the target of interest, CAR-T cells with the ability to activate only upon binding to two or more tumor-associated antigens could markedly improve selectivity. Additionally, chemotherapy or radiation treatment can potentially be administered in lower quantities and smaller periods of time if combined with BsAb and CAR-T therapy, which would lower overall toxicity of site-of-care (SoC) therapies. Overall, improvements in bispecific antibodies and CAR-T therapy and research into new molecular markers in breast cancer, especially TNBC, are of the utmost importance in order to improve outcomes for highly aggressive and metastatic breast cancer. The immunotherapies and selective markers reviewed in this paper will undoubtedly contribute to remarkable advances in cancer therapy.

## Figures and Tables

**Table 1:** Summary of triple-negative breast cancer (TNBC) molecular targets explored by CAR-T cell therapy.

Molecular Target	Cellular/Molecular Function	Tumor Specific (TS) /Tumor Associated (TA)	Malignant Transformation
MUC1 (glycosylated transmembrane protein); tMUC1 (mutated)	Produces mucin; protective barrier against pathogens	TS	Over-expressed and hypoglycosylated; exposure of new protein epitopes
αvβ3 integrin	Promotes migration, invasion, survival, and proliferation	TA	Overexpressed
TEM8 (tumor endothelial marker), ANTXR1	Endothelial cell development	TA	Overexpressed
ROR1 (receptor tyrosine kinase-like orphan receptor 1)	Highly expressed during embryogenesis; promotes migration, invasion, anti-apoptosis	TA	Overexpressed
Mesothelin (glycosylphosphatidylinositol- anchored cell surface receptor)	Migration and invasion	TA	Ove-expressed
AXL (receptor tyrosine kinase)	Cell growth and survival	TA	Overexpressed

**Table 2:** Summary of molecular targets explored by BsAb therapy. All targets are relevant to TNBC unless specified.

Molecular Target	Cellular/Molecular Function	Tumor Specific (TS)/Tumor Associated (TA)	Malignant Transformation
HER2 (transmembrane glycoprotein receptor; epidermal growth factor receptor (EGFR) family); p95HER2 (mutated)	Survival and migration	TS	Truncated version of HER2 only expressed in *HER2+ breast cancer*
VEGFR1 (vascular endothelial growth factor receptor 1)	Receptor tyrosine kinase; Migration, survival, proliferation	TA	Overexpressed
PRLR (prolactin receptor)	Milk production and mammary gland development	TA	Overexpressed
FRα receptor (folate receptor)	Transportation of folate; DNA synthesis biochemical pathway	TA	Overexpressed; BsAb cocktail therapy
EGFR (transmembrane glycoprotein)	Proliferation, adhesion, anti-apoptosis, invasion, angiogenesis	TA	Overexpressed

**Table 3:** Enhancing immunotherapy via modulation of various T cell signals and immune checkpoints.

Immune Checkpoint/T Cell Signal	Function	Therapy Explored in Review
PD-1 (programmed cell death protein 1)	Reduction in pro-survival gene expression within the BCL family, cytokine production, cell cycle progression	CRISPR-CAS9 knockout of PD-1 in CAR-T cells against mesothelin in TNBC
CTLA-4 (cytotoxic T-lymphocyte-associated protein 4)	Inhibit costimulatory signal, inhibit T cell activation by competitively binding CD80 or CD86	mRNA vaccine against MUC1 in TNBC combined with anti-CTLA-4 antibody
LAG-3 (lymphocyte-activation protein 3)	Apoptosis, decreased proliferation of T cells	anti-LAG-3 antibody
TIM-3 (T cell immunoglobulin and mucin domain-containing protein 3)	Reduces immune response in chronic inflammation and tumor infiltrating leukocytes	anti-TIM-3 antibody
TIGIT (T cell immunoreceptor with immunoglobulin and ITIM domains)	Reduces T cell proliferation and cytokine production	BsAb therapy against EGFR in combination with anti-TIGIT-antibody
IL-7R (interleukin 7 receptor)	Proliferation, anti-apoptosis of T cells	Constitutively active IL-7R modified CAR-T cells against AXL
TGF-β (transforming growth factor beta)	Inhibits general functions of immune cell	CAR-T therapy against ROR1 in combination with TGF-β inhibitor (SD-208)

## Data Availability

Data sharing not applicable.

## Abbreviation

CAR-T cell	Chimeric antigen receptor-T cell
BsAb	Bispecific antibody
HER2/ERBB2	Human epidermal growth factor receptor 2
ER	Estrogen receptor
PR	Progesterone receptor
EGFR	Epidermal growth factor receptor
HR	Hormone receptor
TNBC	Triple negative breast cancer
MHC	Major histocompatibility complex
TCR	T cell receptor
scFv	Single-chain variable fragment
APC	Antigen presenting cell
Fab	Fragment antigen binding
PBL	Peripheral blood lymphocytes
BiTE	Bispecific T cell engager (by Amgen)
Fc	Fragment crystallizable
MDA-MB-231	Human breast cancer cell line
HUVEC	Human primary endothelial cell line
Hs578T	Human breast cancer cell line
MDA-MB-468	Human breast cancer cell line
MCF7	Human breast cancer cell line
MDA-MB-435	Human breast cancer cell line
SKBR-3	Human breast cancer cell line
T47D	Human breast cancer cell line
FITC	Fluorescein
antigen 1- antigen 2 BsAb (ex: CD3-p95HER2 BsAb)	Format to describe the two molecular targets of the BsAb
FITC-Folate BsAb (does not follow conventional BsAb naming format)	BsAb targeting FITC and Folate Receptor (folate targets folate receptor)
FITC-DUPA BsAb (does not follow conventional BsAb naming format)	BsAb targeting prostate specific membrane antigen (DUPA targets prostate specific membrane antigen)
FITC-AZA BsAb (does not follow conventional BsAb naming format)	BsAb targeting FITC and carbonic anhydrase (AZA targets carbonic anhydrase)
